# Variation of *Helicoverpa armigera* symbionts across developmental stages and geographic locations

**DOI:** 10.3389/fmicb.2023.1251627

**Published:** 2023-09-07

**Authors:** Chenchen Zhao, Li Wang, Kaixin Zhang, Xiangzhen Zhu, Dongyang Li, Jichao Ji, Junyu Luo, Jinjie Cui

**Affiliations:** ^1^National Key Laboratory of Cotton Bio-breeding and Integrated Utilization, Institute of Cotton Research, Chinese Academy of Agricultural Sciences, Anyang, Henan, China; ^2^Henan International Laboratory for Green Pest Control, College of Plant Protection, Henan Agricultural University, Zhengzhou, Henan, China; ^3^Zhengzhou Research Base, State Key Laboratory of Cotton Biology, Zhengzhou University, Zhengzhou, Henan, China; ^4^Western Agricultural Research Center, Chinese Academy of Agricultural Sciences, Changji, China

**Keywords:** cotton bollworm, development stage, symbionts, geographical population, dynamic change

## Abstract

Cotton bollworm (*Helicoverpa armigera)* poses a global problem, causing substantial economic and ecological losses. Endosymbionts in insects play crucial roles in multiple insect biological processes. However, the interactions between *H. armigera* and its symbionts have not been well characterized to date. We investigated the symbionts of *H*. *armigera* in the whole life cycle from different geographical locations. In the whole life cycle of *H*. *armigera*, Proteobacteria, Firmicutes, Bacteroidetes, and Actinobacteria were the dominant bacteria at the phylum level, while *Enterococcus, Enterobacter, Glutamicibacter*, and *Bacillus* were the four dominant bacteria at the genus level. Furthermore, high similarity in symbiotic bacterial community was observed in different stages of *H*. *armigera*, which were dominated by *Enterococcus* and *Enterobacter*. In fields, the dominant bacteria were Proteobacteria and Bacteroidetes, whereas, in the laboratory, the dominant bacteria were Proteobacteria. At the genus level, the dominant bacteria in cotton bollworm eggs of wild populations were *Enterobacter, Morganella, Lactococcus, Asaia, Apibacter*, and *Enterococcus*, and the subdominant bacteria were *Bartonella, Pseudomonas*, and *Orbus*. Moreover, the symbionts varied with geographical locations, and the closer the geographical distance, the more similar the microbial composition. Taken together, our study identifies and compares the symbiont variation along with geographical gradients and host development dynamic and reveals the high flexibility of microbiome communities in *H. armigera*, which probably benefits for the successful survival in a complicated changing environment.

## Introduction

Host physiological and biochemical functions (development, physiology, ecological interactions, and evolutionary diversity) are closely associated with microbes (Zhu et al., 2022). Host-associated microbiota studies have indicated that microbes can influence host fitness (Jia et al., 2021). In addition, symbionts play key roles in insect development (Hammer et al., 2017). For instance, the nitrogen-recycling activity of gut bacteria in turtle ants (*Cephalotes)* is involved in several biosynthetic pathways contributing to host cuticle formation (Duplais et al., 2021), and burying beetle (*Nicrophorus vespilloides*) preserves carcasses by regulating its microbial growth, which is important for optimal larval development (Shukla et al., 2018). Interestingly, gut microbes of caterpillars can extend to not only just insects but also plant phenotypes and alter outcomes of plant–herbivore interaction (Mason et al., 2019). Deciphering the composition and function of bacterial symbionts and their effects on the hosts poses a significant challenge (Zhao et al., 2019).

The cotton bollworm *Helicoverpa armigera* (Lepidoptera: Noctuidae) is a cosmopolitan pest with a wide host range, and it usually causes huge economic and ecological losses each year. *Helicoverpa armigera* has historically been controlled by transgenic cotton-containing toxic protein from *Bacillus thuringiensis* (Bt) (Downes et al., 2017; Wilson et al., 2018). The Bt cotton planting area has increased considerably in many countries, including China, since 1996 (Cui et al., 2011; Lu et al., 2012). However, in the recent 20 years, Bt cotton planting area contraction in North China Plain has caused the regional resurgence of cotton bollworm, huge crop yield loss, and excess pesticide use (Lu et al., 2022). Considerable studies have demonstrated that symbiont of *H. armigera* can protect the host from baculovirus and Bt biopesticides (Xu et al., 2014; Yuan et al., 2021). However, as a highly polyphagous worldwide lepidopteran pest and an important model system used in various biological research studies, the symbiont profiles and dynamics of *H. armigera* at developmental stages and different geographical locations remain largely unknown.

*Helicoverpa armigera* undergoes complete metamorphosis, and its larvae and adults have distinct morphological characteristics, habitat, feeding behavior, and function. To explore the response of symbionts to host ages and geographical populations, we systematically investigated the bacterial community by 16S ribosomal RNA (16S rRNA) gene sequencing. Our results provide an insight into the interactions between symbionts and *H*. *armigera* in different developmental stages or from multiple cotton planting regions and offer valuable references for further exploring the potential roles of microbiota in host cotton bollworm and developing new management strategies by disturbing bacterial communities.

## Materials and methods

### Insect rearing

**Figure 1:**
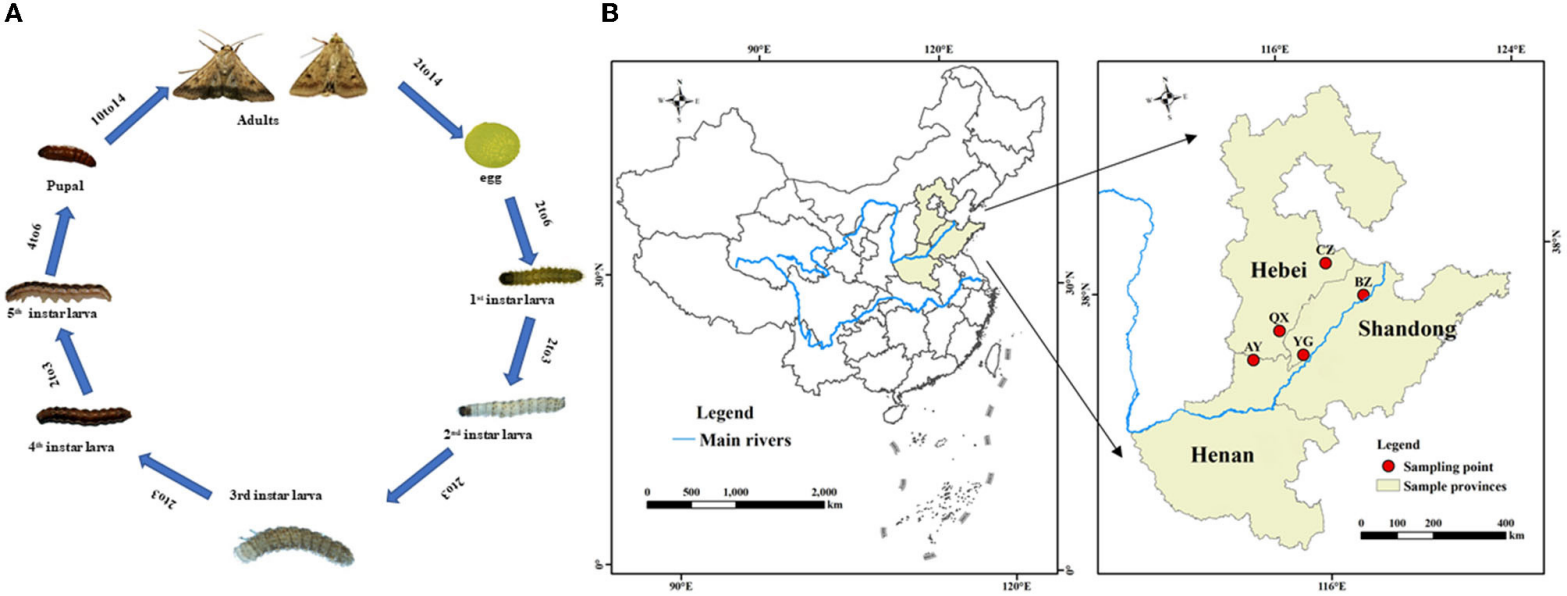
Life cycle **(A)** and a map illustarating regions for sample collection of *Helicoverpa armigera* **(B)**.

Larvae of *H. armigera* were purchased from Henan Jiyuan Baiyun Industry Co., Ltd., Henan, China. Then, *H. armigera* were reared for over three generations without insecticide exposure in the laboratory. Adults were maintained in an environmental chamber and supplied with a 10% honey solution, whereas larvae were maintained on an artificial diet as previously described (Qin, 2015). Rearing conditions were as follows: at 26 ± 1°C, relative humidity of 70 ± 5%, and a 14-h:10-h light: dark cycle. *Helicoverpa armigera* at eight different development stages (eggs, 1st−5th instar larvae, pupae, and adults; Figure 1A) were randomly sampled from the laboratory population. The eight developmental stage experiments of *H. armigera* were performed with three replicates per stage and 20 individuals or 100 eggs per replicate. The samples were immediately flash-frozen in liquid nitrogen and stored at −80°C before DNA extraction.

### Collection of insect field populations

*Helicoverpa armigera* were sampled from six geographical locations in the Yellow River cotton planting region of China (mainly in Henan province, Hebei province, and Shandong province; Figure 1B). Adults were captured with 1,000 W light traps during the second generation.

Field-collected female adults from six locations were placed separately in 250 ml plastic cups covered with cotton gauze on which the female adult laid eggs. A piece of absorbent cotton was placed in each cup containing 10% honey solution for feeding the adults. Female adults were kept at 27–30°C, 70%−80% relative humidity (RH), and light:dark (L:D) = 14:10. We placed the single mated female in a small plastic cup (diameter_1_ = 52 mm, diameter_2_ = 75 mm, and height = 85 mm, covered by sterile plastic paper) for egg collection.

The eggs were collected daily and used for the subsequent experiment. The cotton bollworms captured from each geographical location were divided into five groups, and eggs were collected from each group five times with 300 eggs per time.

### DNA extraction, PCR amplification, and sequencing

Total genomic DNA was extracted from cotton bollworm samples at different development stages or from different geographic populations, respectively, using the TIANamp Genomic DNA Kit (Tiangen Biotech Inc., China), according to the manufacturer's instructions. The surface of the larvae was cleaned with 75% ethanol and rinsed thrice with sterile water before DNA extraction. The lysozyme (50 mg/ml) was added to samples and incubated for 30 min at 37°C, to break up gram-positive bacterial cells. The quantity and quality of the DNA were detected with a NanoDrop 2000C spectrophotometer (Thermo Scientific, USA) and agarose gel electrophoresis, respectively. The V3–V4 variable region of the 16S rRNA gene was amplified using 338F/806R primers (338F: 5′- ACTCCTACGGGAGGCAGCAG-3′, 806R: 5′- GGACTACHVGGGTWTCTAAT-3′) (Xu et al., 2016). The quantification, qualification, and purification of PCR products (Yeasen, China), library preparation, and sequencing were conducted as previously reported (Zhang et al., 2019). Purified amplicons were pooled in equimolar concentration and paired-end sequenced (2 × 300) on an Illumina MiSeq platform (Illumina, San Diego, USA), according to the standard protocols by Shanghai Majorbio Bio-pharm Technology Co., Ltd. The raw reads were submitted to the NCBI Sequence Read Archive (SRA) database with an accession number PRJNA591375.

### Bioinformatics analysis

Data were analyzed as described in previous studies (Zhao et al., 2019, 2020). Based on their unique barcode, paired-end reads were assigned to the library and truncated according to the barcode and primer sequence, to obtain a standard sequence. The obtained sequence reads were processed using QIIME (Caporaso et al., 2010). Partial 16S rRNA bacterial sequences were filtered using Mothur (Schloss et al., 2009), with the inclusion criteria of mean quality score ≥20 and length ≥250 bp. To classify the entire microbial community in *H. armigera*, reads were clustered into operational taxonomic units (OTUs) with a 97% similarity cutoff. Sequence analysis was performed by UPARSE (Edgar, 2013). The rarefied OTU tables were generated to prevent possible sample heterogeneity due to their different sequence numbers. Chao1 and Ace (abundance-based coverage estimator) indices and Shannon and Simpson indices were used to evaluate microbial community richness and diversity, respectively. A Venn diagram was drawn using Venn diagram plotter software (https://omics.pnl.gov/software/venn-diagram-plotter), to visualize unique and shared OTUs across all evaluated samples. Bray–Curtis distance matrices were visualized through principal coordinate analysis (PCoA). UniFrac analysis compared microbial diversity with different samples (Hamady et al., 2010). To investigate community composition differences, we conducted a non-parametric multivariate analysis of variance using the *R* vegan package (Oksanen et al., 2009). Differences in dominant bacteria were analyzed via one-way analysis of variance (ANOVA), followed by Tukey's honestly significant difference (HSD) test using SAS software. Non-parametric Kruskal–Wallis test was performed when data were not normally distributed (*P* < 0.05). These analyses were carried out by Statistics Analysis System software (SAS 9.4, SAS Institute Inc.).

We used Moran's I to analyze the spatial autocorrelation of the dominant bacteria including *Enterobacter, Morganella, Lactococcus, Asaia*, and *Apibacter* in field-collected cotton bollworms. A structural equation model (SEM) (Tenenhaus et al., 2005) with a Satorra–Bentler correction was used to evaluate the effects of geographic or climatic factors on the predominant symbionts in cotton bollworm (Jiang et al., 2023). The temperature and humidity data were provided by the China Meteorological Data Service Center (CMDC) and the global climate database (WorldClim; https://www.worldclim.org/).

### Quantification of bacteria communities

The copies of 16S rRNA genes of 32 dominant bacteria were determined by a previously reported method (Zhang et al., 2019). The standard vector was serially diluted to generate standard curves. The target sequence in the standard vectors was identified by sequencing. The target sequence was amplified through PCR. PCR was performed on a StepOnePlus™ Real-Time PCR System (Applied Biosystems, Foster City, CA, USA). The 20 μl PCR system contained 10 μl 2 × TransStart Green qPCR SuperMix (TransGen Biotech Co., Ltd, China), 0.4 μl each of 10 mM forward and reverse primers, 1.0 μl template DNA (12.5 ng/μl and 2 μl DNA extraction control DNA considered as negative controls), 0.4 μl 50 × ROX, and 7.8 μl H_2_O. The PCR program was as follows: 95°C for 3 min, followed by 40 cycles of a two-step PCR (95°C for 5 s and 60°C for 30 s). Primers used for qPCR analysis are presented in Supplementary Table 1.

## Results

### Overview of *H. armigera* microbiota

Microbial communities in cotton bollworm at different developmental stages or from multiple geographical areas were determined through 16S rRNA sequencing. A total of 1,671 and 1,443 OTUs were identified from cotton bollworm samples of developmental stages or geographical areas, respectively, with an average length of 428 bp (Supplementary Tables 1, 2). These OTUs were clustered into 25 phyla, 548 genera, and 898 species. The rarefaction curves for all samples approached saturation after the number of sequences reached 18,000 (developmental stages) and 35,000 (geographical areas; Supplementary Figure 1), indicating most microbial species were captured in our study. The average sequencing coverage rate of each sample was more than 99.6% (Supplementary Tables 1, 3), which further confirmed that the experimental data accurately reflected the composition of most of the bacterial community.

### Effects of host development stages on microbiome community

**Figure 2:**
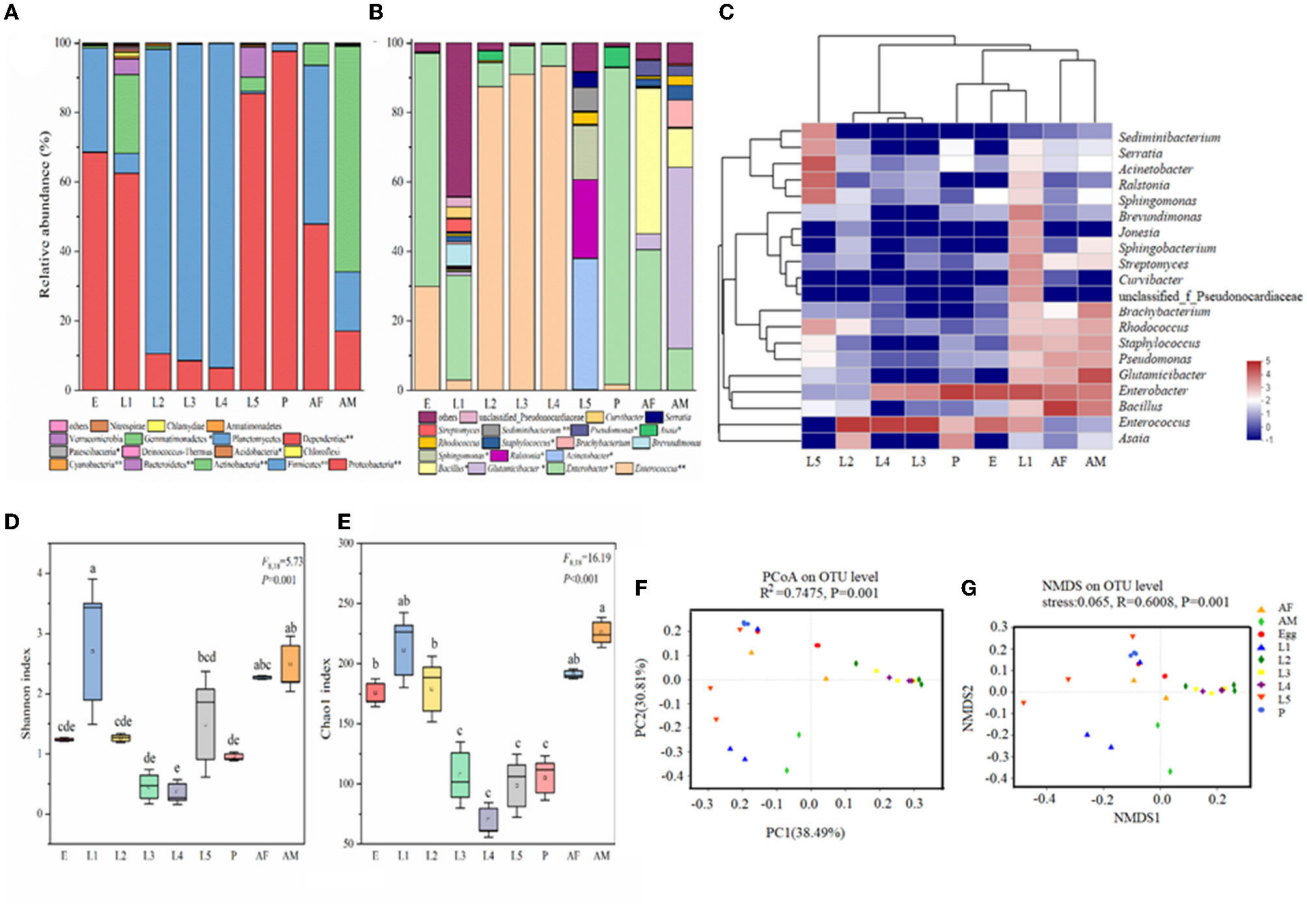
Bacterial community dynamics among different developmental stages in *Helicoverpa armigera*. Relative abundance of bacteria communities at the phylum level **(A)** and genus level **(B)** in different groups (Non-parametric Kruskal–Wallis test *0.01 < *P* ≤ 0.05, **0.001 < *P* ≤ 0.01, and ***P ≤ 0.001). **(C)** Heat map of major taxa over the life cycle at the genus level. Cluster analysis using the Bray–Curtis distance and the complete-linkage method. Each column consisted of top 20 bacteria genus from the eight treatments. Columns are clustered according to the similarity of bacterial abundance profiles. Color gradient represents the proportion of species. Plotting scale, from red to blue, indicates the decrease in richness of bacterial communities. Alpha diversity comparisons of bacterial communities across life stages of *H. armigera* **(D, E)**. Different lowercase labels above each group indicate significant differences (one-way ANOVA, Tukey's HSD test, *P* < 0.05) of group mean value. PCoA plots **(F)** and NMDS plot **(G)** based on weighted-UniFrac of bacterial communities across life stages of *H. armigera*.

Taxonomic analysis showed that Proteobacteria was the most prevalent phylum. Proteobacteria and Firmicutes were the most abundant phyla across all samples (Figure 2A). The relative abundance of Proteobacteria was high in eggs (68.56%), 1st instar larvae (62.46%), 5th instar larvae (85.44%), pupae (97.63%), and female adults (47.89%) and very low in 2nd instar larvae (10.48%), 3rd instar larvae (8.56%), and 4th instar larvae (6.47%). In contrast, the predominant phyla in 2nd, 3rd, and 4th instar larvae were Firmicutes with the relative abundance of 87.73%, 91.04%, and 93.41%, respectively (Figure 2A). At the genus level, there were significant differences in microbial composition and relative abundance at different developmental stages of cotton bollworm (Figure 2B). In contrast to the irregularity of bacteria composition in other developmental stages of *H. armigera*, the 2nd, 3rd, and 4th instar (intermediate instar) larvae exhibited similar microbial community composition, and their dominant bacteria were *Enterococcus* (>87%), which was also dominant in eggs (29.91%). In egg, pupa, female adult, and 1st instar larvae, the dominant bacterium was *Enterobacter* (accounting for 67.09%, 91.26%, 30.22%, and 40.50%). Interestingly, the dominant bacterial genera of the male adult were *Glutamicibacter* (52.26%) and *Bacillus* (11.12%), respectively. The dominant bacteria of the 5th instar larvae were *Acinetobacter* (37.85%), *Ralstonia* (22.62%), and *Sphingomonas* (15.66%; Figures 2B, C).

To further investigate the microbial community present in *H. armigera* larvae, we compared multiple indices of community richness (OTUs, Chao1, ACE, and PD whole tree), diversity (Shannon and Simpson), and sequencing depth (Good's coverage which represents the percentage of the total microbial species detected in a sample; Figures 2D, E, Supplementary Table 1). The average of Good's coverage was estimated to be 99.39%, suggesting that the overwhelming majority of microbial species present in *H. armigera* larvae were included in this study (Supplementary Table 1). The OTU number and Shannon index data showed that the microbiome exhibited large dynamic changes across the eight stages, indicating that *H. armigera* tended to harbor a relatively dynamic microbial diversity. One-way ANOVA showed that the ACE, Chao1, Simpson, and PD tree indices significantly differed across the eight stages (Figures 2C–G). The 1st instar larva and adult displayed higher species richness and diversity than other stages. The microbial community richness in eight stages ranked as follows: 1st instar larvae > male adult > female adult > 5th instar larvae > 2nd instar larvae > egg > pupa> 3rd instar larvae > 4th instar larvae (Figure 2D). The microbial community diversity in eight stages ranked as follows: 1st instar larvae > male adult > female adult > 2nd instar larvae >egg > 3rd instar larvae > pupa > 5th instar larvae > 4th instar larvae (Figure 2E). Taken together, these results indicated that the bacterial community structures in *H. armigera* exhibited dynamic variation across the eight stages.

We performed unconstrained principal coordinate analyses (PCoAs) of UniFrac distances (weighted and unweighted) and Bray–Curtis metrics to investigate patterns of separation among microbial communities in *H. armigera* at different developmental stages (Figures 2C, F, G, Supplementary Table 1). The results showed that the microbiome from cotton bollworm at each of the eight developmental stages clustered separately and exhibited a significant difference in bacterial composition (Adonis, *P* < 0.05; Figures 2F, G). The microbial community composition of the intermediate instar larvae (instars 2–4) was similar, and that of the pupa, egg, and female adult was similar, while that of the 1st instar larvae, 5th instar larvae, and male adult was significantly different from that at other developmental stages (Figure 2F). Complementary analyses were conducted by non-metric multidimensional scaling (NMDS; stress < 0.1, *R* = 0.6008, *P* = 0.001, by ADONIS), which was consistent with those results through PCoA, further indicating the reliability of sample clustering results based on microbial community composition (Figure 2G). The microorganism communities at the 1st instar larvae, the 5th instar larvae, and a male adult significantly differed from those at other instars. The intermediate (2nd to 4th instar) larval microbial communities were similar (Figure 2G). These results suggested significant differences in the microorganism communities of cotton bollworm across different developmental stages.

### Effects of different geographical populations on bacterial community diversity in *H. armigera*

**Figure 3:**
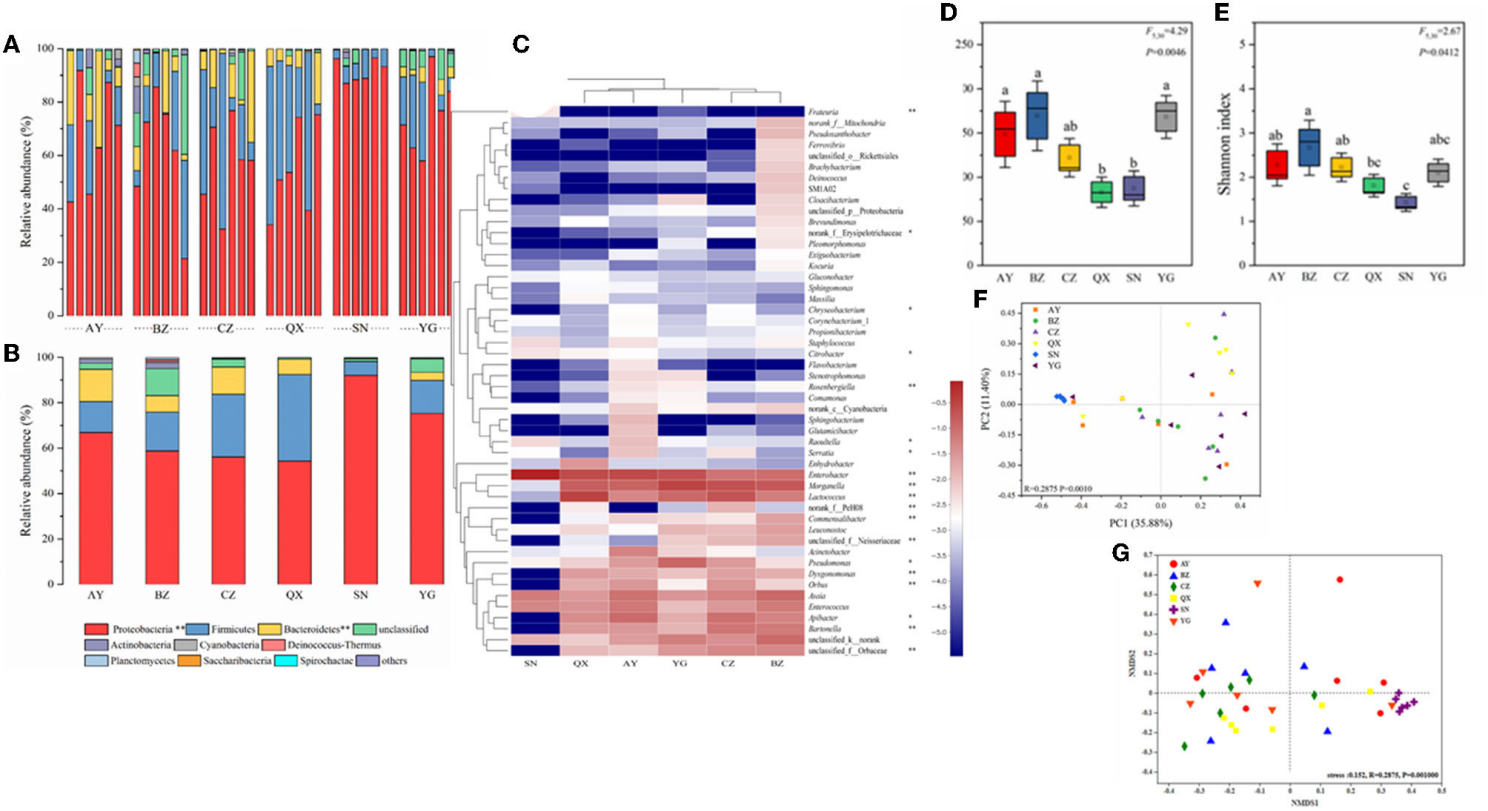
Bacterial community dynamics among different developmental stages in *Helicoverpa armigera*. Relative abundance of bacteria communities at the phylum level **(A, B)** in different groups (Non-parametric Kruskal–Wallis test *0.01 < *P* ≤ 0.05, **0.001 < *P* ≤ 0.01, and ***P ≤ 0.001). Heat map of major taxa over the life cycle at the genus level **(C)**. Cluster analysis using the Bray–Curtis distance and the complete-linkage method. Each column represents top 35 bacteria genus from a certain geographical populations of cotton bollworm and is clustered according to the similarity of bacterial abundance profiles. Boxplot of species richness (number of OTUs) **(D)** and community diversity measured by the Shannon index **(E)**. Different lowercase labels above each group indicate significant differences (one-way ANOVA, LSD *post-hoc* test, *P* < 0.05) of group mean value. PCoA plots **(F)** and NMDS plot **(G)** based on weighted-UniFrac of bacterial communities across life stages of *H. armigera*.

In this study, the richness and diversity of microbial communities in cotton bollworm eggs laid by six field populations and one laboratory population were compared (Figure 3, Supplementary Table 2), with the laboratory population as the control. The results showed that the laboratory population (Lab) exhibited the lowest Sob (observed species) index, followed by the Qiuxian population (QX), which was lower than that of the other three geographical populations, whereas the Binzhou population (BZ) displayed the highest Sob index (*P* < 0.05; Figures 3A, B). The community diversity and species richness of the laboratory population (Lab) were the lowest, followed by the Qiuxian population. Microbial richness increased progressively across the Cangzhou, Anyang, Yanggu, and Binzhou populations but was not significant. Except for the Anyang population, the species richness of geographic populations was inversely proportional to longitude with an order of Qiuxian population < Yanggu population < Cangzhou population < Binzhou population (Figure 3). Simpson, Ace, Chao1, and PD tree indices of the microbial community from these four geographical populations exhibited similar pattern (Supplementary Table 2), which further confirmed the association between the microbial species richness and longitude of host geographic population. The microbial species richness and diversity indices of microbiotas in eggs laid by the laboratory population were also significantly lower than those of field populations (Figures 3D, E, Supplementary Table 2).

To visualize differences in the bacterial community, the histograms of the top nine bacterial genera were constructed among the different sample groups using the QIIME toolkit (Figures 3A, B). According to the OTU representative sequences, Firmicutes, Proteobacteria, Actinobacteria, Bacteroidetes, Cyanobacteria, and Fusobacteria were identified. Firmicutes, Proteobacteria, and Actinobacteria were identified as the dominant community (accounting for >87%) in *H. armigera* at the phylum level (Figures 3A, B).

The heatmap showed the relative abundance of the top 50 genera from six geographical populations. At the genus level, the primary dominant bacteria in cotton bollworm eggs were *Enterobacter, Morganella, Lactococcus, Asaia, Apibacter*, and *Enterococcus*. Their total relative abundance reached 69.13% (Anyang), 54.80% (Binzhou), 68.13% (Cangzhou), 84.66% (Qiuxian), 95.13% (Lab), and 79.97% (Yanggu), respectively. At the genus level, the secondary dominant bacteria were *Bartonella, Pseudomonas*, and *Orbus*. The relative abundance of *Pseudomonas* in the Yanggu population was the highest (9.6%), and the relative abundance of *Bartonella* in the Cangzhou and Binzhou populations (6.4 and 7.99%) was higher than that in other populations (< 3%). The relative abundance of *Acinetobacter* in the Anyang population (5.46%) was higher than that in other geographical populations (< 0.5%). Except for *Enterobacter, Enterococcus*, and *Asaia*, the relative abundance of other bacteria in the laboratory population was extremely low, compared with that of the field population. *Apibacter, Bartonella*, Orbaceae, and *Orbus* were not even detected in the laboratory population (Figure 3C). The cluster analysis showed that the dominant microbial composition in the field populations was similar, the closer the distance was, the higher the similarity was (Anyang–Qiuxian and Cangzhou–Binzhou), and the dominant genus composition in the laboratory population was different. Most of the dominant bacteria in the field populations belong to Proteobacteria and Bacteroidetes, while most of the bacteria in the laboratory population belong to Proteobacteria. In summary, the composition of the microbiome varied greatly across geographic populations.

OTU information of each sample was used to establish the Bray–Curtis distance matrices and draw the PCoA plot, to analyze the differences in microbial composition among six geographic populations (Figure 3F). The results showed that two principal components could explain 35.88 and 11.40% of the total variation. ANOSIM showed that the intra-group differences were smaller than the inter-group differences. PCoA could significantly distinguish laboratory population from field population. The samples of the field populations were separated, indicating that the microbial community structure of the field populations differed greatly within the population. NMDS analysis results (stress < 0.152, *R* = 0.2875, *P* = 0.001) indicated that the grouping was reasonable, and the reliability of this test was high. These results of NMDS analysis were in line with those of PCoA. The laboratory population was relatively clustered and could be distinguished significantly from other populations. The microbial communities of different field populations were interwoven, indicating that the microbial community structure among wild populations was similar (Figure 3G).

**Figure 4:**
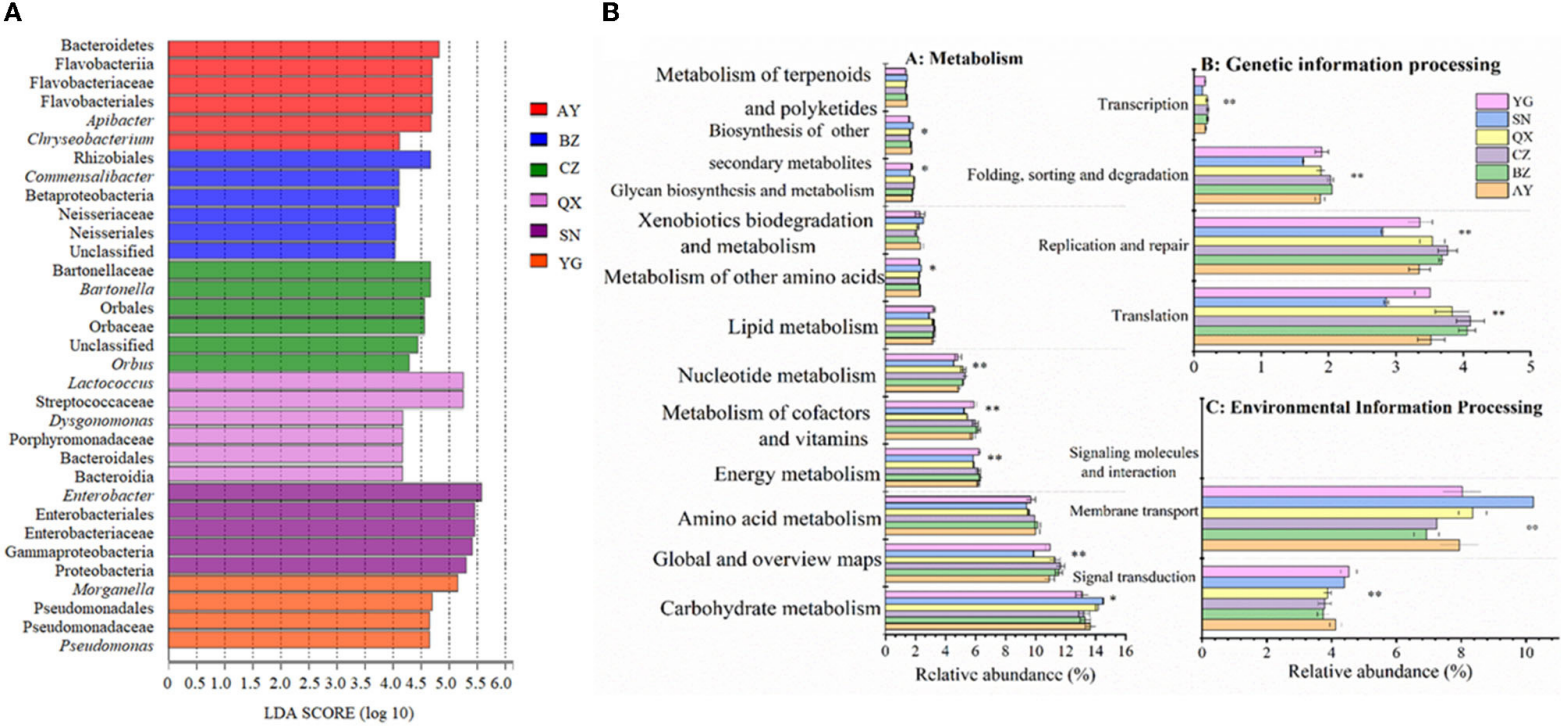
Bacteria of *Helicoverpa armigera* populations with an LDA score of > 4 **(A)** and inferred functions of bacterial communities associated with *H. armigera* in different geographical populations **(B)** (^*^0.05 < *P* ≤ 0.01, ^**^0.05 < *P* ≤ 0.01, and ^***^P ≤ 0.001).

The linear discriminant analysis effect size (LEfSe) was performed to analyze the microbiota of eggs laid by cotton bollworm from different geographical populations (with an LDA threshold of 4; Figure 4A). The result showed that *Bacteroidetes*, Flavobacteriia, Flavobacteriales, Flavobacteriaceae, *Apibacter*, and *Chryseobacterium* were dominant bacteria in the Anyang population. Betaproteobacteria, Rhizobiales, Neisseriaceae, and *Commensalibacter* mainly existed in the Binzhou population. Orbales, Orbaceae, Bartonellaceae, *Bartonella*, and *Orbus* mainly existed in the Cangzhou population. Bacteroidales, Streptococcaceae, Porphyromonadaceae, and *Dysgonomonas* mainly existed in the Qiuxian population. Proteobacteria, Gammaproteobacteria, Enterobacteriales, Enterobacteriaceae, and *Enterobacter* mainly existed in the laboratory population. Pseudomonadales, Pseudomonadaceae, *Morganella*, and *Pseudomonas* mainly existed in the Yanggu population.

Based on the 16S rRNA sequences and the KEGG results, we carried out a phylogenetic investigation of communities by reconstructing unobserved states (PICRUSt), to predict the gene functions of different geographic populations. The results showed that the genes exhibited differences in metabolism, environmental information processing, and genetic information processing (Figure 4B). The relative abundance of symbiotic bacteria involved in carbohydrate metabolism and secondary substance metabolism was significantly higher in the laboratory population than that in the field populations, but the relative abundance of symbiotic bacteria involved in sugar anabolic metabolism, nucleotide metabolism, energy metabolism, and cofactor and vitamin metabolisms was significantly lower in the laboratory population than that in the field populations (*P* < 0.05). Additionally, the relative abundance of symbiotic bacteria involved in lipid and amino acid metabolism was lower in the laboratory population than in the wild populations. The microbial communities in eggs oviposited by wild cotton bollworm populations were more involved in metabolic pathways. The relative abundance of symbiotic bacteria involved in genetic information processing (including translation, replication, repair, folding, classification, degradation, and transcription functions) was significantly lower in the laboratory population than in the wild population (*P* < 0.05). However, the relative abundance of symbiotic bacteria involved in membrane transport and signal transduction (belonging to environmental information processing) was higher in the laboratory population than in the wild population (*P* < 0.05).

### Correlation of environmental factors with the microbial community in *H. armigera*

**Figure 5:**
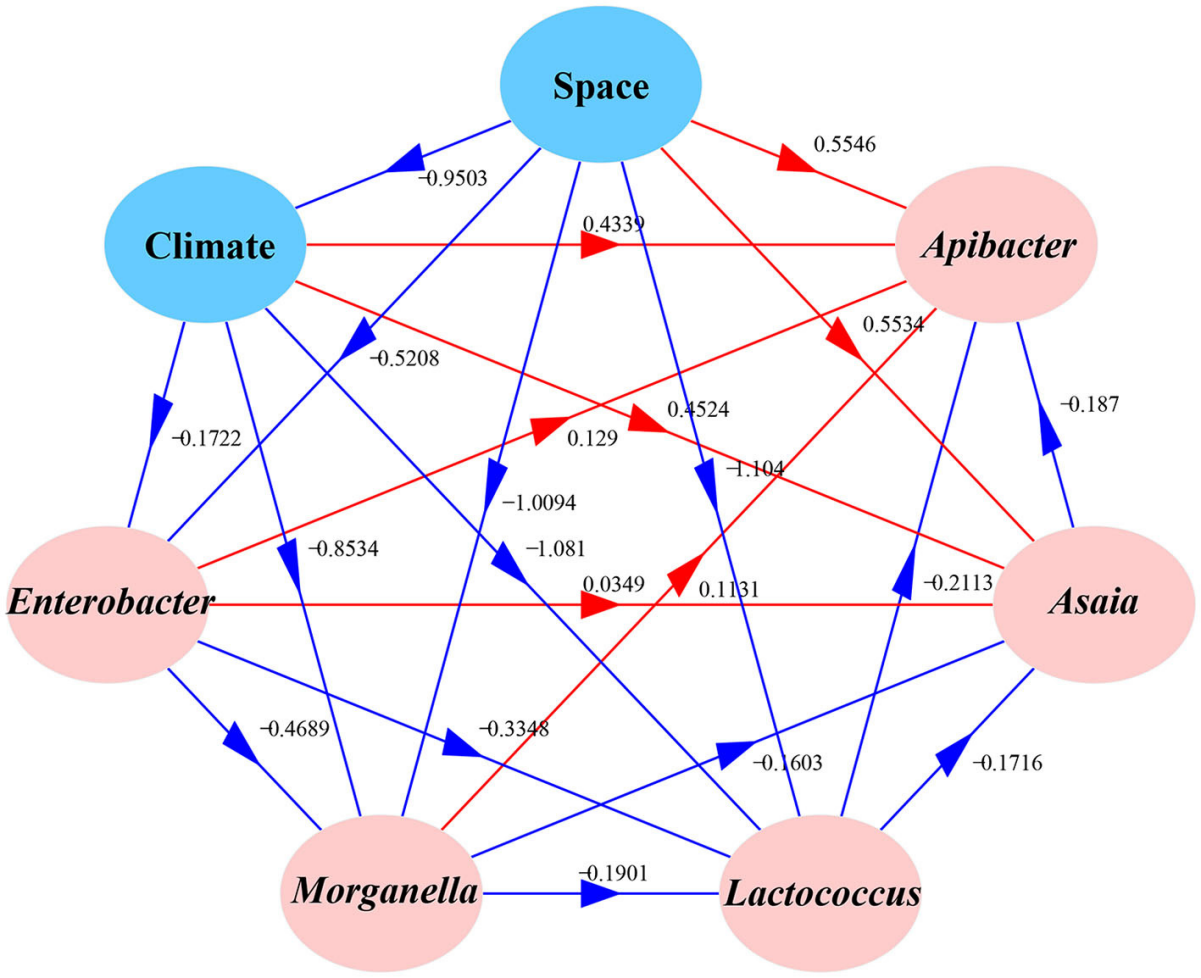
Effects of various factors on the bacterial community compositions of different field populations of *Helicoverpa armigera*. The path diagram is based on the structural equation model (SEM) for environmental factors and microbial Bray–Curtis dissimilarity. The red solid and blue solid lines represent significant positive and negative paths, respectively. The arrow widths represent the strengths of these relationships. The *R*^2^ values of every box indicate the amount of variation in that variable explained by the input arrows. The numbers next to the arrows are unstandardized slopes.

Correlation analysis results showed that *Enterobacter, Morganella, Lactococcus, Asaia*, and *Apibacter* (top 5) were significantly spatially autocorrelated. In addition, the annual mean temperature and precipitation were also significantly spatially autocorrelated (Figure 5). A structural equation model (SEM) explored the relationships between *H. armigera* symbionts and five putative predictive variables (average temperature, precipitation, mean illumination, latitude, and longitude). The results showed that climate factors (temperature, precipitation, and illumination) significantly negatively correlated with space (latitude and longitude; *R* = −0.9503). The relative abundance of *Apibacter* significantly decreased with *Asaia* (−0.187) but increased with space (0.5546) and climate factors (0.4339), and it is also the case with *Asaia*. The proportion of *Enterobacter* significantly decreased with climate factors and space (−0.1722 and −0.9503, respectively), and it was also true with *Morganella* and *Lactococcus*. The proportion of *Asaia* significantly increased with space, climate factors, and other symbionts except *Lactococcus*. Meanwhile, the proportion of *Lactococcus* decreased with the other four symbionts, space, and climate factors.

### RT-qPCR analysis of the top 32 bacteria

**Figure 6:**
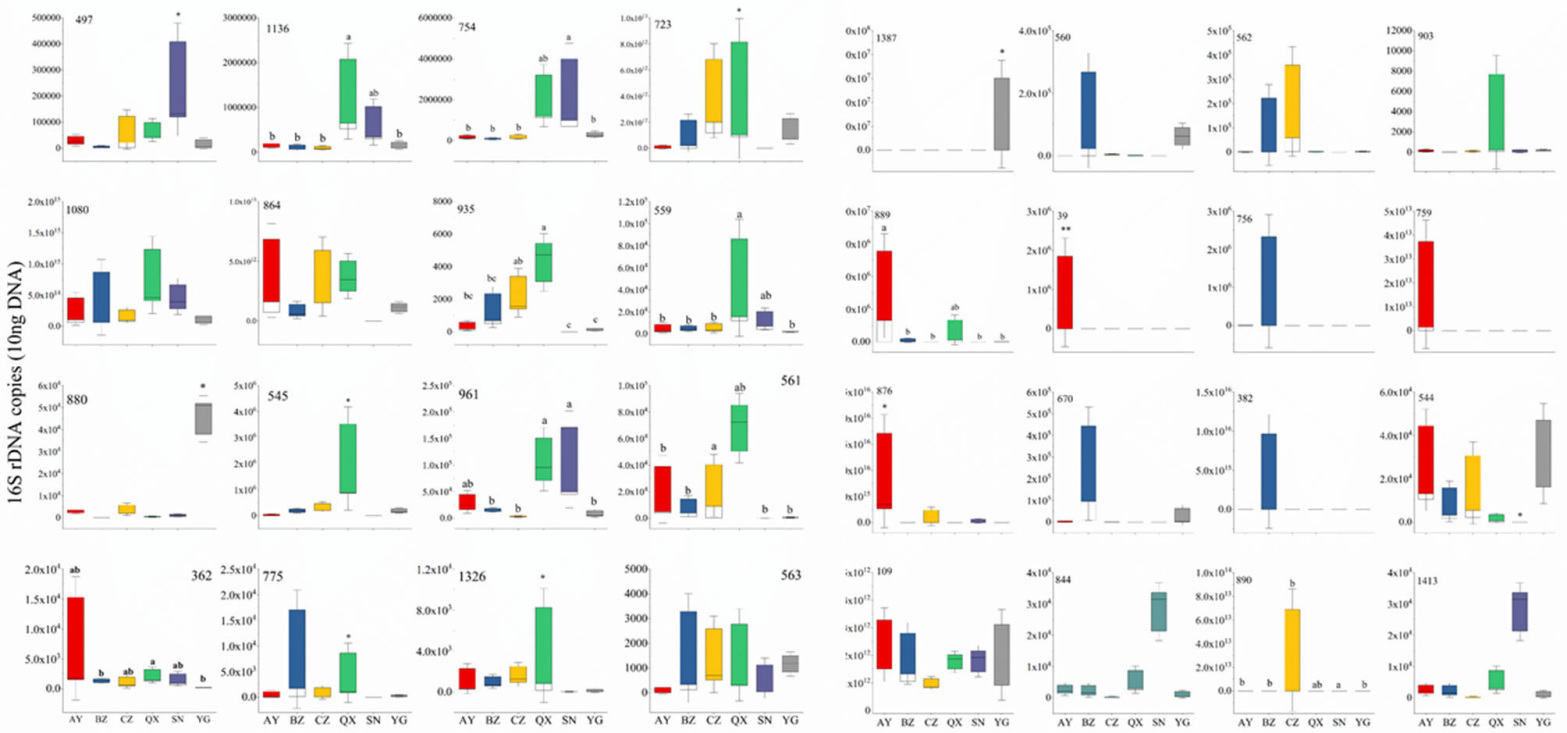
Thirty-two widespread bacterial species out of total bacterial 16S rDNA gene copies by quantitative PCR. Different lowercase labels above each group and ^*^ indicate significant differences (one-way ANOVA, Tukey's HSD test, *P* < 0.05) of group mean value.

To evaluate bacterial response to geographical locations, the top 32 bacterial genera (relative abundance) were selected for RT-qPCR. All the bacterial sequences were aligned to the NCBI database, and specific primers of the top 32 bacteria were designed by DNAMAN (Supplementary Table 4). The qPCR results showed that *Serratia* rarely appeared in samples, and *Enterococcus* appeared in the egg of *H. armigera*. Most OTUs were not significantly different between indoor and wild *H. armigera* larvae populations at the same stage. Copy numbers of OTU1387 (*Bartonella*), 890 (*Citrobacter*), 889 (*Dysgonomonas*), 39 (*Acinetobacter*), 876 (*Serratia*), 544 (*Rosenbergiella*), 497 (*Enterobacter*), 1,136 (*Morganella*), 754 (*Lactococcus*), 723 (*Lactococcus*), 935 (*Bartonella*), 559 (*Enterococcus*), 880 (*Pseudomonas*), 545 (*Rosenbergiella*), 961 (*Acinetobacter*), 561 (*Orbus*), 362 (*Enterococcus*), 1,326 (*Enterococcus*), and 563 (*Pseudomonas*) in the laboratory population were significantly different from those in the wild population, respectively (Figure 6). *Apibacter, Lactococcus, Morganella, Bartonella, Orbus, Dysgonomonas*, and *Commensalibacter* were not detected in the laboratory population, while they were widely present in the wild population. RT-qPCR results indicated significant differences in bacterium copy numbers among different geographical populations.

## Discussion

In China, *H. armigera* is one of the most destructive pests in cotton (Wu and Guo, 2005), and Bt cotton has been an effective measure to control this pest since 1997. The percentage of Bt cotton increased from 50% in 2000 to almost 100% in 2004 in Yellow River, China (Zhang et al., 2011; Lu et al., 2012). The Yellow River region was once the largest cotton-planting area, and Henan, Shandong, and Hebei provinces were the main cotton-growing provinces in this region. In the Yellow River region, wheat is the main host plant of first-generation *H. armigera* larvae, and subsequent generations mainly feed on cotton, maize, peanuts, and soybeans (Wu and Guo, 2005).

Insects inhabit different ecological niches and face challenges such as adverse food sources, extreme environments, and threats from natural enemies. Interestingly, bacterial symbionts can promote insect fitness, and these beneficial effects of bacterial symbionts on insects' response to environmental stress can be attributed to strong selective overlap between the microbiota and their insect host from an evolutionary perspective (Douglas, 2015). The interaction between plants and phytophagous insects is driven by a hidden interplay between their microbiota (Di Lelio et al., 2023). *Helicoverpa armigera* is a worldwide migrant pest with highly variable distances traveled under different circumstances (Jones et al., 2019). Hence, the evaluations of the potential effects of bacterial symbionts on its host at different stages from different geographic regions are crucial for this destructive pest.

In this study, four phyla of bacteria (Proteobacteria, Firmicutes, Actinobacteria, and Bacteroidetes) and two genera of bacteria (*Enterobacter* and *Enterococcus*) dominated across all life stages of cotton bollworm, which was similar to several other lepidopteran pests (Chen et al., 2018). At the phylum level, there were no significant differences in the microbiota composition of the cotton bollworm across the whole life cycle from different geographical populations, which might be due to the simple intestinal structure of lepidopteran pests (Engel and Moran, 2013). These microbiota have been reported to play a vital role in insect physiology and metabolism (Jones et al., 2013). At the genus level, microbiota composition varied among developmental stages and geographic regions. Environmental habitat, food, and phylogeny of the host affected microbial community structure at different developmental stages (Yun et al., 2014; Staudacher et al., 2016; Zhao et al., 2019). Indeed, food can significantly alter the composition of host microbes (Wu et al., 2011). The insect–microbe interaction may improve the insect's ability to adapt to its environment and explore new niches. Microbial colonization and stable interactions are common in insects and can be mutualistic (Pais et al., 2018). The resistance of cotton bollworm to Bt is related to the contents of *Enterobacter* and *Enterococcus* (Broderick et al., 2006, 2009). *Enterobacter* can reduce the mortality of larvae of *Plutella xylostella* after exposure to Bt protein (Raymond et al., 2009). Typical intestinal bacteria such as *Streptococcus* spp. and *Staphylococcus* spp. have strong influences on the growth of *B. thuringiensis* in *Homona magnanima* larvae (Takatsuka and Kunimi, 2000). Hence, the high abundance of *Enterococcus* and *Enterobacter* in wild cotton bollworm populations probably has contributed to the increased resistance to Bt in recent 20 years. In addition, the diversity and variability of symbiotic bacteria in cotton bollworm might also explain its strong adaptability, wide distribution, and broad host range.

*Enterobacter* and *Enterococcus* are the top symbiotic bacteria in Lepidoptera (Broderick et al., 2004; Teh et al., 2016). Interestingly, these two bacteria are also found in the microbiota of the cotton bollworm eggs, indicating that they might be probiotics and can be vertically transmitted. The high abundance of *Enterobacter* in egg and pupa might be because neonates or moths often consume eggshells upon hatching or eclosion (Duplouy and Hornett, 2018), and *Enterobacter* contributes to the increase in the host fitness (Augustinos et al., 2015). In addition, lepidopteran pests such as *H. armigera* typically consume their chorions after hatching to obtain symbiotic bacteria to form microbiota rapidly to meet their own growth and development needs (Chen et al., 2018).

Insects' symbionts exhibit correlations with environmental changes across diverse geographical regions, responding to shifts in local ecological factors such as temperature, precipitation, altitude, and interactions with natural adversaries. The diversity of bacteria among psyllid species has been associated with their host plant affiliations and geographic dispersion, demonstrating robust preservation of Enterobacteriaceae secondary symbionts across distinct regions (Martoni et al., 2023). Within the realm of stingless bees, as the geographical span between colonies widens, the resemblance between them diminishes for both species, as observed in the study by Liu H. et al. (2023). The application of Mantel tests brought noteworthy associations between geographic proximity and symbiont communities in two specialized aphid species (Xu et al., 2020). An intriguing observation is the reliance of caterpillar microbiomes on soil microbiomes. This enables the transmission of plant-induced soil effects to subsequent insects feeding on different plants, as highlighted by Hannula et al. (2019). Notably, the highest bacterial genetic diversity in topsoil is found in temperate habitats, where the composition of microbial genes exhibits greater variation in response to environmental factors than mere geographical distances, an insight unveiled by Bahram et al. (2018). In our investigation, a positive correlation emerged between temperature and geographical distance. The resemblance in microbial composition within field populations, exemplified by Anyang–Qiuxian and Cangzhou–Binzhou, might find its explanation in climate-related latitude factors, offering a novel perspective on the dynamics of microbial communities.

Insect gut microbial diversity is determined by various factors including its environmental habitat, diet, developmental stage, and host phylogeny (Martinson et al., 2017; Chen et al., 2018). In this study, the highest microbial diversity was observed in the initial and final developmental stages (egg and adult). However, the bacterial diversity was significantly lower in the larva and pupa stages of *H. armigera* than in its egg and adult stages. This microbial diversity difference associated with development stages is common in lepidopterans since they undergo holometabolous metamorphosis, in which they entirely reshape the body structure and possess an unusually alkaline gut environment with an ~2-h rapid food intake (Paniagua Voirol et al., 2018). The insect population's susceptibility to plant resistance may be attributed to gut microbiota composition (Liu M. et al., 2023). Microorganisms are key players in the modulating of insect physiological pathways required to face the challenge when consuming plants (Di Lelio et al., 2023). The microorganism–host interactions further increase the complexity of host bio-evolution (Rosenberg and Zilber-Rosenberg, 2016). Hence, diet and development stages majorly influence bacterial community variability (Staudacher et al., 2016).

One study of systemic symbiosis in jewel wasps has demonstrated that the composition and function of the microbial communities are closely related to host evolution (Brooks et al., 2016). To complete their life cycle, the insects could utilize the energy supply from their symbiotic bacteria. Bacteria in the lepidopteran gut such as *Enterobacter* spp. can provide ROS-detoxifying enzymes such as superoxide dismutase or catalase (Xia et al., 2017). However, the roles of highly abundant *Enterobacter* in cotton bollworm remain to be further explored.

This study indicated that the dominant symbiotic bacteria determined the microbial community composition difference in cotton bollworm eggs of various geographical populations. Gene function prediction analysis showed that the relative abundance of microbiotas involved in translation, replication and repair, folding, classification, degradation, and transcription functions was significantly lower in the laboratory population than in the wild population, indicating that the field population of cotton bollworm could be better adapted to various environment stresses (such as plant defense, pathogenic bacteria, fungi, and virus, and insecticides), which benefited from the symbiotic bacteria. As a return, environmental factors also have a great impact on the microbial community composition. The richness and diversity of the microbial community in the laboratory population were significantly lower than in the field population. Furthermore, the closer the geographical distance was, the more similar the habitat climate and crop composition were, and the greater the similarity in microbial composition among hosts from geographically close areas. Similarly, the microbial communities of hosts from both field population and laboratory population have been investigated in several fruit flies such as *Poecilus chalcites, Anopheles stephensi*, and *Ostrinia nubilalis*, and microorganisms in the field population exhibited high diversity but low relative abundance with even distribution (Lehman et al., 2009; Rani et al., 2009; Belda et al., 2011; Chandler et al., 2011). The microbial community composition of *Lymantria dispar* varies significantly with various foods (Mason and Raffa, 2014). In the larvae of *Heliothis virescens*, the microbiota in the indoor and wild populations differed greatly and did not share any OTUs (Staudacher et al., 2016). A major reason for the low diversity of microbial communities in the laboratory insect populations might be that artificial foods often contain antibiotics that reduce and/or alter the diversity of microbes in the insects (Tang et al., 2012).

In addition, cotton bollworm is omnivorous and can come into contact with corn, wheat, and other crops in the wild (Wu and Guo, 2005), and the shell of eggs produced by field populations carries more microorganisms from the adult and can transfer to larvae due to the neonates' habit of feeding egg shell after hatching (Koyle et al., 2016; Kietz et al., 2018). Food can significantly influence host microbial diversity (Wu et al., 2011; Yun et al., 2014). Host plant species have been reported to have a greater impact on midgut bacterial diversity than the geographical location of the host plant, and the larval midgut symbionts were similar to those of food leaves (Priya et al., 2012). In this study, the microbial diversity difference in cotton bollworm eggs from different geographic populations may result from the combined effects of habitat environment and the food taken by the mother generation. *Commensalibacter, Bartonella, Orbus, Dysgonomonas, Morganella, Pseudomonas*, and other bacteria exhibited high abundance in cotton bollworms from different geographic populations. Thus, they may be the dominant bacteria contributing to the field populations' adaptation to the local environment. We speculated that the geographical environment could directly affect the diversity of microorganisms in insects and indirectly affect their progenies.

## Conclusion

We show that variations in food, developmental stages, and geographical locations all affect the composition of bacterial communities of this lepidopteran. The microbiota in *H. armigera* exhibits dynamic composition. The symbiont communities of the different stages of *H. armigera* differed greatly from each other. Close geographical proximity leads to more similar microbial composition, likely due to similar climate and crop patterns. Altogether, these discoveries highlight how developmental and environmental cues dramatically reshape symbiotic bacterial communities in *H. armigera*, a globally destructive invasive species. The findings illuminate the underpinnings of microbial interactions in this pest, laying the groundwork to unravel symbiont functional roles and devise novel microbiome-based management strategies that disrupt associations optimizing its fitness and adaptability. Much remains to be discovered about the tripartite cross-talk between host, microbiota, and habitat sustaining the success of this agricultural nemesis.

## Data availability statement

The datasets presented in this study can be found in online repositories. The names of the repository/repositories and accession number(s) can be found at: https://www.ncbi.nlm.nih.gov/, PRJNA591375.

## Author contributions

JC, JL, and JJ carried out lab work. CZ and JC participated in data analysis and drafted the manuscript. LW, KZ, XZ, and DL participated in data analysis. CZ conceived of the study, designed the study, coordinated the study, and helped draft the manuscript. All authors gave final approval for publication.

## Supplementary material

Supplementary Figure 1 Rarefaction curves of bacterial communities across life cycle **(A)** and from different geographic population **(B)** of *H. armigera*.

Supplementary Table 1 Summary of high-throughput sequencing read analysis, bacterial community diversity richness (OTUs, 97%), sample coverage (Good's coverage), diversity index (Shannon, ACE, Shannon, Simpson), and estimated OTU richness (Chao1) for community diversity analyses of 27 samples from *H. armigera* in different development stages.

Supplementary Table 2 Summary of high-throughput sequencing read analysis, bacterial community diversity and richness index in different geographic population of *H. armigera*.

Supplementary Table 3 Number of different taxonomic categories of bacterial communities across whole *H. armigera* life cycle.

Supplementary Table 4 Primer sequences used in this experiment.
