# Supplementary figures and images for: Variation of Helicoverpa armigera symbionts across developmental stages and geographic locations
Source: Front Microbiol. 2023 Sep 7;14:1251627. doi: 10.3389/fmicb.2023.1251627 (PMC10513443; doi:10.3389/fmicb.2023.1251627)

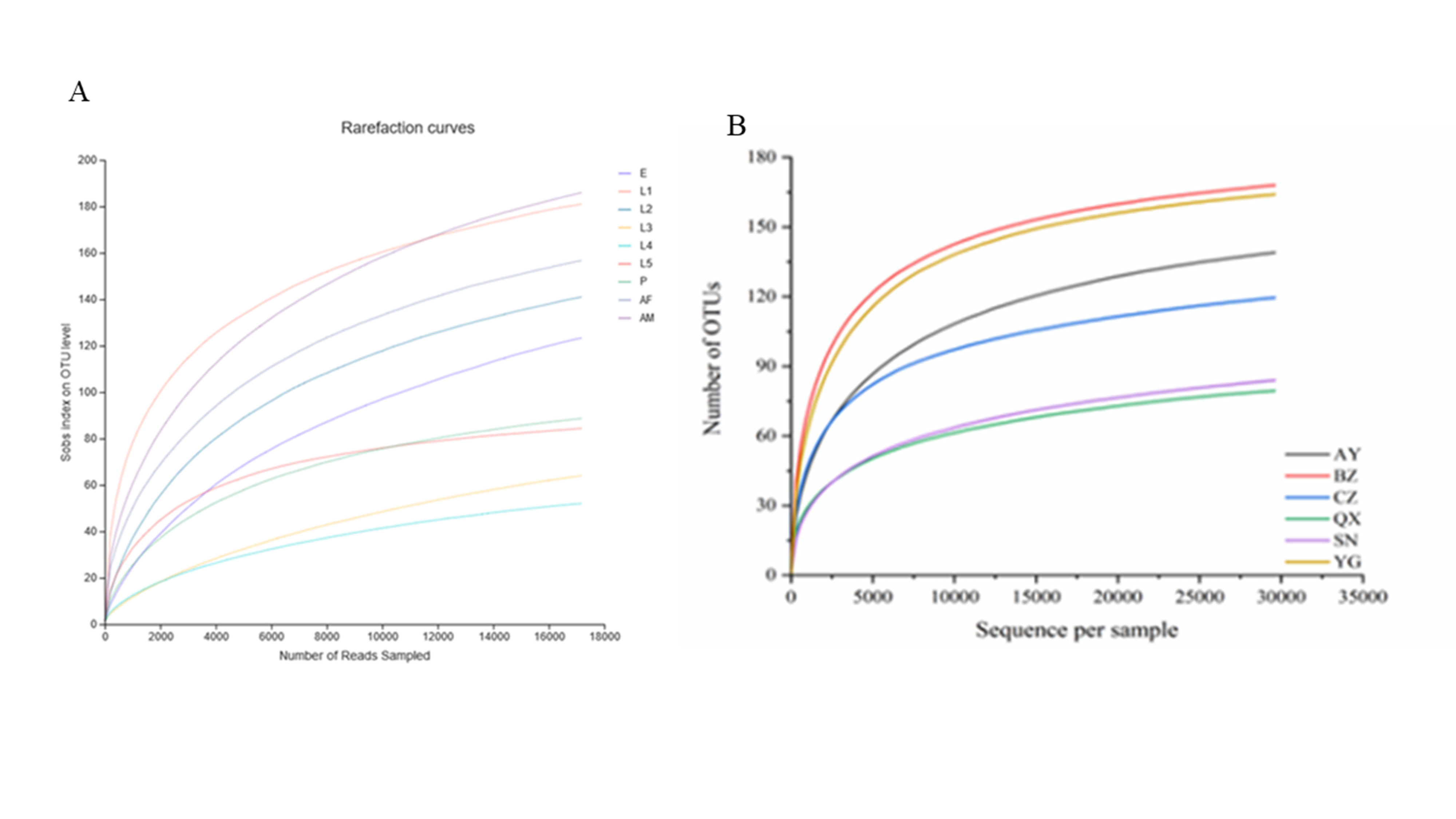

Supplement: Supplementary Figure 1 — Rarefaction curves of bacterial communities across life cycle (A) and from different geographic population (B) of H. armigera. [file Image_1.tif]
