# Supplementary material for: Variation of Helicoverpa armigera symbionts across developmental stages and geographic locations
Source: Front Microbiol. 2023 Sep 7;14:1251627. doi: 10.3389/fmicb.2023.1251627 (PMC10513443; doi:10.3389/fmicb.2023.1251627)
Supplement: Supplementary Table 1 — Summary of high-throughput sequencing read analysis, bacterial community diversity richness (OTUs, 97%), sample coverage (Good's coverage), diversity index (Shannon, ACE, Shannon, Simpson), and estimated OTU richness (Chao1) for community diversity analyses of 27 samples from H. armigera in different development stages. [file Table_1.docx]

**Supplementary table 1 Summary of high-throughput sequencing read analysis, bacterial community diversity richness （OTUs, 97%）, sample coverage （Good’s coverage）, diversity index （Shannon, ACE, Shannon, Simpson）, and estimated OTU richness （Chao1） for community diversity analyses of 27 samples** **from *H. armigera* in different development stages.**

| Sample information | Seq_num | Base_num | Mean_length | Diversity index | | | | | |
| --- | --- | --- | --- | --- | --- | --- | --- | --- | --- |
|  |  |  |  | Sobs | Shannon | Simpson | ACE | Chao1 | Coverage |
| E1 | 51951 | 22261361 | 428.51 | 137 | 1.2018 | 0.4185 | 183.63 | 191.05 | 99.73% |
| E2 | 34359 | 14741401 | 429.04 | 110 | 1.2296 | 0.3431 | 227.34 | 167.65 | 99.70% |
| E3 | 48934 | 20995404 | 429.06 | 123 | 1.2719 | 0.3392 | 181.03 | 168.54 | 99.70% |
| L1_1 | 60893 | 25431025 | 417.63 | 213 | 3.5749 | 0.0815 | 228.37 | 226.14 | 99.86% |
| L1_2 | 76750 | 31886082 | 415.45 | 213 | 3.4333 | 0.0759 | 238.91 | 237.23 | 99.79% |
| L1_3 | 108961 | 46724485 | 428.82 | 117 | 1.0904 | 0.4374 | 208.68 | 170.26 | 99.71% |
| L2_1 | 46249 | 19760341 | 427.26 | 121 | 1.2682 | 0.4023 | 141.69 | 143.56 | 99.83% |
| L2_2 | 44953 | 19249763 | 428.22 | 180 | 1.3407 | 0.4294 | 208.31 | 204.38 | 99.77% |
| L2_7 | 84092 | 35938635 | 427.37 | 122 | 1.1715 | 0.4512 | 294.28 | 188.50 | 99.67% |
| L3_1 | 33432 | 14360632 | 429.55 | 66 | 0.7728 | 0.6122 | 113.09 | 101.43 | 99.81% |
| L3_2 | 56273 | 24187387 | 429.82 | 63 | 0.1120 | 0.9743 | 172.44 | 141.75 | 99.79% |
| L3_3 | 49461 | 21253727 | 429.71 | 63 | 0.4719 | 0.8101 | 91.26 | 78.81 | 99.87% |
| L4_1 | 42514 | 18269558 | 429.73 | 64 | 0.6311 | 0.7268 | 118.75 | 89.00 | 99.85% |
| L4_2 | 39588 | 17010311 | 429.68 | 46 | 0.2678 | 0.9125 | 66.06 | 59.91 | 99.90% |
| L4_3 | 50953 | 21906025 | 429.93 | 46 | 0.1877 | 0.9456 | 76.99 | 61.11 | 99.90% |
| L5_1 | 54012 | 23201800 | 429.57 | 39 | 0.3468 | 0.8845 | 56.29 | 65.25 | 99.91% |
| L5_2 | 33316 | 13822982 | 414.91 | 97 | 2.2658 | 0.1550 | 104.18 | 106.10 | 99.92% |
| L5_3 | 47834 | 20221557 | 422.74 | 117 | 1.8599 | 0.3029 | 124.13 | 124.00 | 99.91% |
| P_1 | 44374 | 18997478 | 428.12 | 101 | 1.0457 | 0.5622 | 122.47 | 122.00 | 99.84% |
| P_2 | 53117 | 22660892 | 426.62 | 97 | 0.9148 | 0.6227 | 119.11 | 111.77 | 99.85% |
| P_3 | 35715 | 15293569 | 428.21 | 68 | 0.9027 | 0.6238 | 77.92 | 81.13 | 99.91% |
| AF_1 | 95568 | 41052118 | 429.56 | 151 | 2.2929 | 0.2066 | 183.49 | 190.00 | 99.77% |
| AF_2 | 42883 | 18367110 | 428.31 | 154 | 2.2848 | 0.2091 | 197.61 | 187.44 | 99.75% |
| AF_3 | 42883 | 18367110 | 428.31 | 165 | 2.2263 | 0.2501 | 196.67 | 196.54 | 99.76% |
| AM_1 | 85584 | 36657544 | 428.32 | 173 | 2.1975 | 0.2333 | 223.13 | 224.00 | 99.70% |
| AM_2 | 43336 | 18500853 | 426.92 | 213 | 3.1046 | 0.0794 | 245.03 | 241.29 | 99.74% |
| AM_3 | 30172 | 12894730 | 427.37 | 172 | 2.1780 | 0.2359 | 222.07 | 212.55 | 99.72% |
