# Supplementary material for: Variation of Helicoverpa armigera symbionts across developmental stages and geographic locations
Source: Front Microbiol. 2023 Sep 7;14:1251627. doi: 10.3389/fmicb.2023.1251627 (PMC10513443; doi:10.3389/fmicb.2023.1251627)
Supplement: Supplementary Table 2 — Summary of high-throughput sequencing read analysis, bacterial community diversity and richness index in different geographic population of H. armigera. [file Table_2.docx]

**Supplementary table 2 Summary of high-throughput sequencing read analysis, bacterial community diversity and richness index in different geographic population** **of *H. armigera***

| Sample Information | Seq_num | Base_num | | Mean_length | Diversity index | | | | | |  |
| --- | --- | --- | --- | --- | --- | --- | --- | --- | --- | --- | --- |
|  |  |  |  |  | Sobs | Shannon | Simpson | ACE | Chao1 | pd | Coverage |
| AY_1 | 30355 | 13444582 | | 442.91 | 123 | 2.2991 | 0.1558 | 137.85 | 139 | 34.28 | 99.92% |
| AY_2 | 31773 | 14087065 | | 443.37 | 95 | 1.7745 | 0.2397 | 111.62 | 108 | 21.28 | 99.93% |
| AY_3 | 37439 | 16535713 | | 441.67 | 225 | 2.9870 | 0.1152 | 245.77 | 266 | 75.64 | 99.91% |
| AY_4 | 34798 | 15570118 | | 447.44 | 71 | 1.4237 | 0.3013 | 79.77 | 77 | 23.33 | 99.96% |
| AY_5 | 35774 | 15865897 | | 443.50 | 191 | 1.7917 | 0.3995 | 203.67 | 202 | 84.38 | 99.93% |
| AY_6 | 33365 | 14773317 | | 442.78 | 186 | 3.4065 | 0.0775 | 188.09 | 190 | 22.06 | 99.98% |
| BZ_1 | 37044 | 15677931 | | 423.22 | 219 | 4.0763 | 0.0293 | 224.34 | 228 | 88.66 | 99.97% |
| BZ_2 | 36213 | 15825783 | | 437.02 | 226 | 2.7538 | 0.1282 | 233.53 | 237 | 101.21 | 99.94% |
| BZ_3 | 34058 | 14780731 | | 433.99 | 111 | 1.7310 | 0.3547 | 147.22 | 146 | 38.78 | 99.90% |
| BZ_4 | 34305 | 15326294 | | 446.77 | 90 | 1.3033 | 0.4223 | 96.54 | 96 | 28.77 | 99.96% |
| BZ_5 | 31310 | 13867851 | | 442.92 | 137 | 2.8525 | 0.0830 | 146.60 | 143 | 51.05 | 99.94% |
| BZ_6 | 45317 | 18787002 | | 414.57 | 234 | 3.2819 | 0.1177 | 239.14 | 247 | 104.45 | 99.97% |
| CZ_1 | 31671 | 14090952 | | 444.92 | 108 | 2.1499 | 0.2029 | 127.42 | 147 | 22.38 | 99.92% |
| CZ_2 | 33322 | 14928436 | | 448.01 | 109 | 2.0152 | 0.2220 | 133.53 | 133 | 34.63 | 99.91% |
| CZ_3 | 39452 | 17673331 | | 447.97 | 78 | 1.4452 | 0.4160 | 96.14 | 102 | 19.40 | 99.95% |
| CZ_4 | 31262 | 13829211 | | 442.36 | 145 | 2.6835 | 0.1113 | 148.99 | 150 | 36.06 | 99.97% |
| CZ_5 | 31450 | 13448985 | | 427.63 | 180 | 2.9362 | 0.1222 | 190.10 | 199 | 79.99 | 99.94% |
| CZ_6 | 36608 | 16223945 | | 443.18 | 112 | 2.1155 | 0.1940 | 134.93 | 145 | 31.95 | 99.93% |
| QX_1 | 40510 | 18191080 | | 449.05 | 50 | 1.5653 | 0.3400 | 101.47 | 76 | 12.01 | 99.96% |
| QX_2 | 41073 | 18438586 | | 448.92 | 61 | 1.4902 | 0.3238 | 78.44 | 73 | 16.73 | 99.96% |
| QX_3 | 36225 | 16081816 | | 443.94 | 130 | 2.2281 | 0.2023 | 135.63 | 137 | 45.35 | 99.97% |
| QX_4 | 41044 | 18273400 | | 445.21 | 92 | 2.4103 | 0.1456 | 108.56 | 106 | 26.17 | 99.95% |
| QX_5 | 37277 | 16702655 | | 448.07 | 76 | 1.4022 | 0.3877 | 93.70 | 89 | 23.71 | 99.95% |
| QX_6 | 34436 | | 15374041 | 446.45 | 89 | 1.7637 | 0.2566 | 98.78 | 102 | 39.75 | 99.96% |
| SN_1 | 37341 | | 16790267 | 449.65 | 84 | 1.0324 | 0.4591 | 97.64 | 97 | 18.75 | 99.95% |
| SN_2 | 32493 | | 14510549 | 446.57 | 126 | 1.9416 | 0.3218 | 129.87 | 144 | 42.28 | 99.97% |
| SN_3 | 33725 | | 15033300 | 445.76 | 127 | 1.6821 | 0.3673 | 132.28 | 134 | 58.71 | 99.96% |
| SN_4 | 41480 | | 18577099 | 447.86 | 57 | 1.3362 | 0.3437 | 117.74 | 107 | 24.75 | 99.94% |
| SN_5 | 43869 | | 19510135 | 444.74 | 76 | 1.3179 | 0.3422 | 102.67 | 109 | 16.38 | 99.94% |
| SN_6 | 47239 | | 21199310 | 448.77 | 54 | 1.2528 | 0.3848 | 62.91 | 61 | 5.22 | 99.97% |
| YG_1 | 36078 | | 15935835 | 441.71 | 168 | 2.1855 | 0.2390 | 176.31 | 184 | 68.80 | 99.95% |
| YG_2 | 31017 | | 13760749 | 443.65 | 199 | 1.9686 | 0.3276 | 209.73 | 212 | 79.63 | 99.92% |
| YG_3 | 39641 | | 17584199 | 443.59 | 161 | 2.4485 | 0.1537 | 181.10 | 186 | 60.03 | 99.93% |
| YG_4 | 42303 | | 19016793 | 449.54 | 95 | 1.2198 | 0.4614 | 103.41 | 102 | 7.57 | 99.96% |
| YG_5 | 40541 | | 17697356 | 436.53 | 182 | 2.0919 | 0.3633 | 191.95 | 198 | 72.34 | 99.95% |
| YG_6 | 30635 | | 13643315 | 445.35 | 204 | 2.6887 | 0.2192 | 206.20 | 205 | 68.62 | 99.98% |
