# Supplementary material for: Variation of Helicoverpa armigera symbionts across developmental stages and geographic locations
Source: Front Microbiol. 2023 Sep 7;14:1251627. doi: 10.3389/fmicb.2023.1251627 (PMC10513443; doi:10.3389/fmicb.2023.1251627)
Supplement: Supplementary Table 3 — Number of different taxonomic categories of bacterial communities across whole H. armigera life cycle. [file Table_3.docx]

**Supplementary table 3 Number of different taxonomic categories of bacterial communities across whole *H. Armigera* life cycle**

| Samples | Phylum | Class | Order | Family | Genus | Species | OTU |
| --- | --- | --- | --- | --- | --- | --- | --- |
| E | 16(0) | 38(2) | 82(4) | 120(5) | 190(9) | 262(17) | 399(29) |
| L1 | 20(4) | 50(9) | 117(24) | 223(63) | 441(148) | 711(284) | 1075(500) |
| L2 | 11(0) | 21(0) | 61(1) | 104(3) | 185(3) | 151(4) | 472(128) |
| L3 | 12(1) | 23(1) | 54(1) | 68(1) | 98(2) | 136(3) | 226(5) |
| L4 | 8(0) | 9(0) | 19(0) | 25(0) | 35(0) | 50(0) | 115(0) |
| L5 | 17(1) | 29(3) | 68(8) | 107(13) | 188(32) | 243(54) | 279(74) |
| P | 13(0) | 21(0) | 44(0) | 65(13) | 96(0) | 125(1) | 225(2) |
| AF | 10(0) | 13(0) | 33(1) | 54(2) | 93(4) | 131(5) | 367(38) |
| AM | 13(1) | 22(1) | 57(1) | 100(5) | 183(18) | 257(27) | 481(67) |
| Total | 25[7] | 60[8] | 140[13] | 264[11] | 548[8] | 898[8] | 1671[8] |

**Note: () represents only in this stage, [] represents shared in each stage.**
