# Supplementary material for: Variation of Helicoverpa armigera symbionts across developmental stages and geographic locations
Source: Front Microbiol. 2023 Sep 7;14:1251627. doi: 10.3389/fmicb.2023.1251627 (PMC10513443; doi:10.3389/fmicb.2023.1251627)
Supplement: Supplementary Table 4 — Primer sequences used in this experiment. [file Table_4.docx]

**Supplementary Table 4 Primer sequences used in this experiment**

| OTU | Phylum | Sense（5’-3’） | Anti-Sense（5’-3’） |
| --- | --- | --- | --- |
| 497 | Proteobacteria | GAGGAAGGCGATAAGGTTA | CACATCCGACTTGACAGA |
| 1136 | Proteobacteria | AGCCATGCCGCGTGTATGAAG | CGGTGCTTCTTCTGTCGGTAACG |
| 723 | Firmicutes | GGAATCTTCGGCAATGGA | GGCTGCTGGCACGTAGTTAG |
| 754 | Proteobacteria | AGCCATGCCGCGTGTATGAAG | GGAGTTAGCCGGTGCTTCTTCTG |
| 1080 | Proteobacteria | CGGAATGACTGGGCGTAA | GTCAGTAATGAGCCAGGTTGC |
| 864 | Bacteroidetes | TGAGAGCCTGATCCAGCCATCC | CGCTTGCATCCTCCGTATTACCG |
| 935 | Proteobacteria | GCTCAACCCTGGAACTGCCTTT | GCACCTCAGCGTCAGTAATGGA |
| 559 | Firmicutes | CGTAGGTGGCAAGCGTTGTCC | CGCCTTCGCCACTGGTGTTC |
| 880 | Proteobacteria | GGTGCAAGCGTTAATCGG | TTCGCCACTGGTGTTCCT |
| 545 | Proteobacteria | GGAAGGTAGCAATTCTAACAG | CATCTCAGCGTCAGTCTT |
| 961 | Proteobacteria | TTACCCGCAGAAGAAGCACC | TTCACATCCGACTTGACAGACC |
| 561 | Proteobacteria | GAGTATTGTAGAGGAAGGTAGA | GCATCTCAGCGTCAGTAT |
| 362 | Firmicutes | TCGGCAATGGACGAAAGT | GGCTGCTGGCACGTAGTTAG |
| 775 | Bacteroidetes | ACGCAAGTCTGAACCAGCCAAG | CGCTCGCATCCTCCGTATTACC |
| 1326 | Firmicutes | TTCCACCCTTGACGGTATCTAA | CAGTTTCCAATGACCCTCCC |
| 563 | Firmicutes | TTCGGGTCGTAAAGCACTGTTG | CGGATAACGCTCGGGACATAC |
| 1387 | Proteobacteria | GAATTTCCTGTGTAGCGGTG | GGGTTTCTAATCCTGTTTGC |
| 560 | Proteobacteria | GAGTGTGTCAGAGGGGGGT | GGGTTTCTAATCCTGTTTGCT |
| 562 | Proteobacteria | CTGGTTATCTGGAGTCTTGT | CATCTCAGCGTCAGTCTT |
| 903 | Proteobacteria | GGTGTAGCGGTGAAATGCG | TAGGGTTTCTAATCCTGTTTGC |
| 889 | Bacteroidetes | TTCCAGTTTCAACGGCAA | GGTAATACGGAGGATGCG |
| 39 | Proteobacteria | AATACTCTTGGATAGTGGACG | CTACGCACGCTTTACGCC |
| 756 | unclassified_k__norank | TCATCAGGAATGTAGCCA | TGTGAATGTACTTAACCGA |
| 759 | Proteobacteria | TGAGTCTTGTAGAGGGGGG | GTCAGTCTTTGTCCAGGGG |
| 876 | Proteobacteria | GTTACTCGCAGAAGAAGCACC | AGTTCCCAGGTTGAGCCC |
| 670 | unclassified_k__norank | GTAGTCTATGAGTGCGGG | GGTAGATACTCTCTTGCCA |
| 382 | Proteobacteria | GAAACTGCGGGAAATCGG | TGCTGGGAAGAAGTGGGGC |
| 544 | Proteobacteria | GTGTAGCGGTGAAATGCG | GGGTTTCTAATCCTGTTTGCT |
| 109 | Actinobacteria | ATTCCTGGTGTAGCGGTGA | TGGGGTATCTAATCCTGTTCG |
| 844 | unclassified_k__norank | CAGGAGGCGGCATAAACT | TGCGATGAACTACAGCGA |
| 890 | Proteobacteria | GTTACTCGCAGAAGAAGCACC | ACATCCGACTTGACAGACCG |
| 1413 | Proteobacteria | TAAGTCAAATGTGAAATCCCC | TGCCTTCGCCATCGGTAT |
